# Within‐family relations of mental health problems across childhood and adolescence

**DOI:** 10.1111/jcpp.13572

**Published:** 2022-01-24

**Authors:** Lydia Gabriela Speyer, Hildigunnur Anna Hall, Yuzhan Hang, Claire Hughes, Aja Louise Murray

**Affiliations:** ^1^ Department of Psychology University of Edinburgh Edinburgh UK; ^2^ 2152 Department of Psychology University of Cambridge Cambridge UK

**Keywords:** Parental mental health, internalising, externalising, within‐family, Millennium Cohort Study

## Abstract

**Background:**

While transactional models suggest that parent and child mental health reciprocally influence one another over development, research has largely focused on parent‐to‐child effects. Additionally, it is not known whether observed associations hold when appropriate statistical tools are used to operationalise within‐family dynamics.

**Methods:**

We investigated within‐family mental health dynamics using autoregressive latent trajectory models with structured residuals, stratified by child gender. Parental psychological distress was assessed using the Kessler (K6) scale, and children’s internalising and externalising problems were assessed using the Strengths and Difficulties Questionnaire. Both measures were administered at the age 3, 5, 7, 11, 14 and 17 waves of the Millennium Cohort Study (*N* = 10,746, ~50% female).

**Results:**

Maternal psychological distress was positively associated with subsequent internalising and externalising problems for girls but only with internalising problems for boys. Paternal psychological distress was associated with boys’ later internalising and externalising problems during early adolescence. Among boys, internalising problems were associated with later maternal psychological distress, while externalising problems were associated with later paternal psychological distress. Among girls, internalising problems were associated with subsequent paternal psychological distress, while externalising problems were associated with later maternal psychological distress. Finally, maternal and paternal psychological distress showed negative bidirectional associations in early childhood but positive associations in middle childhood and early adolescence.

**Conclusions:**

Findings support a transactional model of family mental health, with both child‐to‐parent and parent‐to‐child effects playing a role in the development of mental health difficulties. Mental health intervention efforts should, therefore, target the whole family system.

## Introduction

Maternal mental health difficulties, such as symptoms of anxiety or depression, are well‐established risk factors for childhood mental health problems (e.g. Baker, Devine, Ng‐Cordell, Raymond, & Hughes, 2020; Goodman, 2020; Gotlib, Goodman, & Humphreys, 2020), including externalising problems (e.g. conduct problems) (Dora & Baydar, 2019) and internalising problems (e.g. depression) (Gotlib et al., 2020). Potential explanations include transmission of genetic risk factors, impacts of prenatal maternal depression on foetal neurodevelopment, dysfunctional parenting and insecure attachment (Goodman, 2020). Several studies have highlighted that familial factors such as genetic risks and shared family environment explain considerable variance in the associations between child and maternal mental health (e.g. Gjerde et al., 2021).

In transactional models of child development (e.g. Sameroff & Chandler, 1975), parent–child transactions contribute to associations between parent and child mental health, with children viewed as active participants in shaping their social environment, including their parents’ behaviour and mental health. However, existing research has mostly focused on how maternal mental health problems affect child mental health. The few studies to address bidirectional effects have yielded mixed findings: child‐driven effects on parental psychopathology are reported in some studies (Baker, Devine, et al., 2020; Tyrell, Yates, Reynolds, Fabricius, & Braver, 2019; Xerxa et al., 2021) but not others (Choe, Olson, & Sameroff, 2014; Gross, Shaw, Moilanen, Dishion, & Wilson, 2008). Moreover, despite meta‐analytic evidence suggesting effects of paternal mental health problems on child adjustment problems, especially externalising problems (Connell & Goodman, 2002), most investigations of parent–child transactions focus on maternal mental health problems, leaving fathers comparatively overlooked (Bögels & Phares, 2008). Including fathers is also important for investigating the reciprocal impacts of mental health between parents. Recent studies to examine reciprocal relations between child, paternal and maternal mental health (e.g. Tyrell et al., 2019; Xerxa et al., 2021) have yielded mixed findings, highlighting the need for further research in this area.

Notably absent are longitudinal investigations: existing studies of relations between child and parental mental health rely heavily on cross‐sectional data, precluding analyses of directional effects. Studies adopting a longitudinal approach have mostly focused on just two time points (e.g. age 2 and age 4; Gross et al., 2008); or a limited developmental span (e.g. early childhood; Baker, Devine, et al., 2020; or adolescence; Tyrell et al., 2019); or employed long time intervals between measurement occasions (e.g. age 3 and age 10; Xerxa et al., 2021); or only investigated parent‐to‐child effects (Flouri, Sarmadi, & Francesconi, 2019).

Another methodological limitation of existing research relates to the statistical operationalisation of dynamics that are presumed to play out *within* families. Most previous studies have relied on comparing group‐level differences, that is between‐person differences in mental health problems that reflect the effects of time‐stable risk factors differing across families (e.g. the effects of genes). However, the developmental effects of primary interest in transactional theories reference within‐person and within‐family processes. For example, increased paternal mental health problems may be hypothesised to lead to increases child internalising problems within the same family. Illuminating these within‐family mechanisms can yield more suitable targets for intervention (Hamaker, Kuiper, & Grasman, 2015).

Previous longitudinal research has commonly used methods such as the cross‐lagged panel model (e.g. Baker, Brooks‐Gunn, & Gouskova, 2020). However, these models do not appropriately disentangle within‐ from between‐family effects and so yield ambiguous results with regard to the within‐family dynamics most commonly of interest in developmental theories of child–parent mental health transactions (Berry & Willoughby, 2017). Two exceptions deserve note. Tyrell et al. (2019) tracked 392 Mexican–American and European–American adolescents (ages 13, 15, 20 and 22 years), using a trait and time‐varying cross‐lagged model to investigate within‐family relations of parental and adolescent mental health. Xerxa et al. (2021) tracked 5,536 Dutch children (1.5, 3 and 10 years), using an autoregressive latent trajectory model with structured residuals (ALT‐SR) to investigate family mental health dynamics. Both studies reported reciprocal relations between parent and child mental health.

Bridging the developmental scope of these two studies, the current study uses data from the Millennium Cohort Study, a large UK representative birth cohort study (*N* = 10,746) to explore the within‐family developmental relations between both maternal and paternal mental health problems, such as symptoms of anxiety and depression, and child internalising and externalising problems across childhood and adolescence (ages 3, 5, 7, 11, 14 and 17). Rather than using a clinical sample, we focus on a general population sample. Although this limits our ability to identify parent–child mental health transactions as they play out for those with serious mental health issues, our community‐ascertained sample has some advantages. This includes being less likely than a clinically ascertained sample to be influenced by Berkson’s bias (i.e. overestimation of symptom co‐occurrence due to different symptoms independently affecting treatment seeking; Berkson, 1946) or range restriction (i.e. underestimation of symptom co‐occurrence due to participants only showing symptoms on the upper extreme; Murray, McKenzie, Kuenssberg, & O’Donnell, 2014). Meaningful variation in mental health problems exists both below and above clinical cut‐offs (Conway & Krueger, 2021), making it important to study parent–child mental health relations in representative samples that include the full spectrum of continuously distributed mental health symptoms. We focus on child internalising and externalising as key outcomes as these are the most common mental health issues experienced over this developmental period (e.g. Ghandour et al., 2019).

To appropriately operationalise within‐family dynamics, we built an autoregressive latent trajectory model with structured residuals (ALT‐SR) to disaggregate within‐ and between‐family effects. As associations between parental and child mental health may differ between boys and girls (e.g. Tyrell et al., 2019; Xerxa et al., 2021), we stratified by gender. There is limited research to guide specific hypotheses regarding effects at particular developmental stages and involving parents and children of particular genders. Based on transactional models that assume reciprocal effects, we investigated both parent‐to‐child and child‐to‐parent effects and considered whether these span the entirety of child and adolescent development, involve both mothers and fathers and hold for both boys and girls.

## Methods

### Participants

Participants were children and parents taking part in the Millennium Cohort Study (MCS), a longitudinal birth cohort study that used a stratified sampling procedure to follow around 19,000 children born in the United Kingdom between September 2000 and January 2002. Socially disadvantaged families and families living in areas with high ethnic minority concentration were intentionally oversampled to ensure the sample is representative of the UK population (Connelly & Platt, 2014; Joshi & Fitzsimons, 2016). Design weights, attrition weights, stratification variables and clustering variables are provided to account for the complex sampling design and nonrandom dropout. The first wave of data collection took place when children were aged 9 months; subsequent waves took place at ages 3, 5, 7, 11, 14 and 17 years. To ensure that our sample was as representative as possible of the general population, the current study included all children who participated up to age 17 (*N* = 10,746, female = 5,376, male = 5,370), irrespective of whether they grew up in a single parent household or with two parents. Additional analyses with families that consisted of a single parent household throughout the study period are reported at: https://osf.io/st32q/?view_only=6222430c80374ad688ec6e2cdec18f73. For sample demographic information at baseline, see Table S1.

### Measures

#### Child mental health

The Strengths and Difficulties Questionnaire (SDQ) (Goodman, 1997) includes five domains: prosociality, peer problems, emotional problems, conduct problems and ADHD symptoms. All subscales consist of five items, rated on a 3‐point Likert‐type scale (‘not true’, ‘somewhat true’, ‘certainly true’). Item scores are summed (range: 0–10) with higher scores indicating more problem behaviours or more prosociality. The peer problem and emotional problem subscales can be summed to form a broader internalising domain (range: 0–20), while conduct problems and ADHD symptoms form a broader externalising domain (range: 0–20). In the MCS, the SDQ was predominantly completed by mothers who rated their child’s behaviour at ages 3, 5, 7, 11, 14 and 17. At age 3, an age‐adapted version of the SDQ was administered. Psychometric analyses of the SDQ have generally been favourable (for a review, see Kersten et al., 2016), showing invariance across informants, gender and development (ages 5–14) in the MCS (Murray, Speyer, Hall, Valdebenito, & Hughes, 2021a, 2021b).

#### Parental mental health

During the same data collection waves as children’s internalising and externalising behaviours, maternal and paternal mental health was measured using the Kessler (K6) Scale. The K6 is a widely‐used brief screening tool for mental health problems in the general population that focuses on symptoms of depression and anxiety (Kessler et al., 2003). It consists of six items (see Appendix S1) that assess general psychological distress in the past 30 days. These items (e.g. ‘How often did you feel hopeless?’) scored on a 5‐point Likert‐type scale ranging from ‘none’ to ‘all of the time’ are summed to provide a total score for psychological distress (range: 0–24). The K6 scale predicts psychiatric disorders in the general population (Kessler et al., 2010) and has good psychometric properties, including good internal reliability in the study sample (Flouri et al., 2019).

### Statistical analysis

Using Mplus 8.5 (Muthén & Muthén, 2018), ALT‐SRs were fitted separately for boys and girls to investigate the within‐family dynamics in maternal, paternal and child mental health. ALT‐SRs combine a parallel process latent growth curve model with a cross‐lagged panel model. The latter part can be used to investigate reciprocal effects between residuals created from the growth curve part of the model. These residuals represent time‐specific deviations from the person‐specific growth curves. As the growth curve part captures between‐person differences in baseline levels of mental health difficulties and partials these out of the cross‐lagged part of the model, an ALT‐SR distinguishes within‐family from between‐family effects (Curran, Howard, Bainter, Lane, & McGinley, 2014).

We included random intercept factors for each mental health construct and allowed these to covary. Including the random intercept covariances is critical because it allows the model to account for temporally stable factors (i.e. time‐invariant confounders) such as shared genes and stable aspects of the shared family environment that may be associated with between‐person differences in multiple mental health problems and thus account for their between‐person associations (Mund, Johnson, & Nestler, 2021). Slope factors were fixed, and their loadings were set proportional to the distances between measurement waves for linear slopes and to the square of these distances for quadratic slopes. For parental mental health, linear growth curves were estimated, whereas for child mental health, growth curves included a quadratic term since previous research has shown that children’s mental health trajectories follow a curvilinear trajectory across childhood and adolescence (e.g. Murray, Eisner, Nagin, & Ribeaud, 2020). Cross‐lagged as well as autoregressive paths were defined between the residuals from the parallel process growth curve part of the model.

Models were fit using robust maximum likelihood estimation (MLR) with full information maximum likelihood (FIML), thus addressing missing data. Models were adjusted for the complex sampling design of the MCS by including attrition weights, stratification variables and clustering variables. Including attrition weights helps correct for nonrandom dropout by deriving weights based on an individual’s estimated probability of responding and using these weights to up‐weigh observations from those unlikely to respond relative to those likely to respond. Models were judged to have acceptable fit if the Tucker Lewis Index (TLI) and Comparative Fit Index (CFI) were >.90 and Root Mean Squared Error of Approximation (RMSEA) was <.05 (Kline, 2005). Considering the large number of tests conducted, we report both unadjusted and adjusted p‐values based on the generalised Holm *k*‐familywise error rate (FWER) correction. *K*‐FWER is less conservative than traditional FWER corrections that can lead to a substantial loss of statistical power, and hence type‐2 errors, when many statistical tests are conducted (Keselman, Miller, & Holland, 2011). In *K*‐FWER, *k* represents the probability of rejecting at least *k* hypotheses H*i* with *i* representing one hypothesis of a set of true null hypotheses. *K* is chosen based on the number of statistical tests of interest conducted (in our case 120 tests representing all cross‐lagged parameters) multiplied by *alpha* (.05), resulting in a *k* of 6.

As noted above, the ALT‐SR should account for unmeasured time‐invariant confounders. However, as a sensitivity analysis we also fit the model adjusting for the following baseline covariates: maternal age, maternal education (no qualification/qualification), Index of Multiple Deprivation (as indicator of socio‐economic status), presence of siblings (yes/no), maternal smoking during pregnancy (yes/no), gestational age and child ethnicity (white/other).

We then tested whether these time‐invariant confounders affected our within‐person estimates. First, we fit models for both male and female children, assuming stable effects on our outcomes (e.g. assuming that maternal smoking during pregnancy has equal effects on a child’s mental health at age 3 and at age 14) by regressing the random intercept factors on all baseline covariates. As this assumption of stable effects many not always hold, we then fit models assuming time‐varying effects on our outcomes. To test this, we directly regressed the observed outcome variables at each time point (e.g. internalising problems at age 5) on the baseline factors. Forcing these parameters to be equal at each time point equates to a model that regresses only the random intercept factors on the covariates. Thus, we can compare the model assuming *time‐invariant covariates with stable effects* to a model assuming *time‐invariant covariates with time‐varying effects*. By examining the models’ Bayesian Information Criterion (BIC) values, we tested the assumed stable effects of baseline factors (Mund et al., 2021). Lower values on the model assuming *time‐invariant covariates with stable effects* indicate that the assumption of stable effects holds. Missing data on covariates was dealt with using multiple imputation. For Mplus code and full model results, see: https://osf.io/st32q/?view_only=6222430c80374ad688ec6e2cdec18f73.

## Results

Table S2 summarises descriptive statistics, including measures of internal consistency.

### Boys subsample

**Figure 1:**
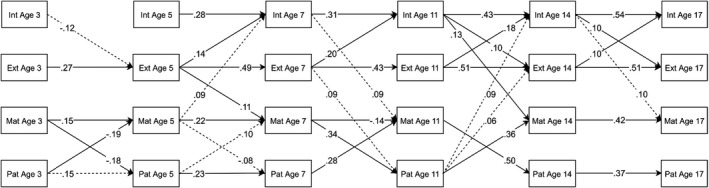
Standardised autoregressive and cross‐lagged parameters from the ALT‐SR for boys. Int = internalising, Ext = externalising, Mat = maternal psychological distress, Pat = paternal mental health. Only statistically significant paths are shown. Dotted lines are significant based on *p* < .05. Solid lines are significant based on *q* < .05. Latent growth curve and covariance parameters are omitted for clarity. Ages correspond to children’s median ages at data collection.

The ALT‐SR for boys fit well (RMSEA = .033, CFI = .950, TLI = .915). Figure 1 summarises significant standardised autoregressive and cross‐lagged parameters. For full results, see Tables S3, S4a and S5a.

Maternal psychological distress at age 5 was associated with within‐child increases in internalising problems at age 7. Paternal psychological distress at age 11 was associated with within‐child increases in internalising problems at age 14. However, these associations were not significant after correcting for multiple comparisons. From middle childhood, internalising problems showed consistent within‐family cross‐lagged effects on maternal mental health with the age 11 to age 14 association remaining significant after corrections. Externalising problems were associated at the within‐family level with increased maternal psychological distress at age 7 and paternal psychological distress at age 11; however, this effect was not significant after applying *k‐*FWER corrections. Finally, from age 7 to age 11 and age 11 to age 14, paternal and maternal psychological distress showed bidirectional within‐family associations, with maternal psychological distress being associated with paternal psychological distress and vice versa.

### Girls subsample

**Figure 2:**
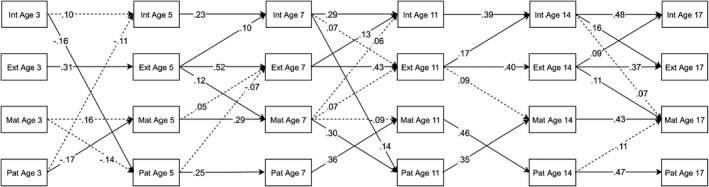
Standardised autoregressive and cross‐lagged parameters from the ALT‐SR for girls. Int = internalising, Ext = externalising, Mat = maternal mental health, Pat = paternal mental health. Only statistically significant paths are shown. Dotted lines are significant based on *p* < .05. Solid lines are significant based on *q* < .05. Latent growth curve and covariance parameters are omitted for clarity. Ages correspond to children’s median ages at data collection.

For girls, the ALT‐SR showed good fit (RMSEA = .036, CFI = .944, TLI = .905) with parental psychological distress steadily increasing across childhood and adolescence while internalising and externalising problems declined at first but increased later on. Figure 2 provides significant standardised autoregressive and cross‐lagged parameters. For full results, see Tables S4b, S5b and S6.

In contrast to the ALT‐SR for boys, externalising problems were associated with increased maternal psychological distress during both childhood and adolescence. Internalising problems, however, were only associated with increased maternal psychological distress at age 17, and this effect was not robust to multiple comparisons corrections. Regarding paternal psychological distress, internalising at age 11 was associated with increased paternal psychological distress at age 14, whereas from age 3 to age 5, internalising problems were associated with decreased paternal psychological distress. Paternal psychological distress at age 5 had a negative cross‐lagged effect on age‐7 externalising problems, while maternal psychological distress at age 5 had a positive cross‐lagged effect on age‐7 externalising and was further associated with increased internalising and externalising problems from age 7 to age 11. However, these effects were all nonsignificant after applying *k‐*FWER corrections.

### Sensitivity analysis

Results of the sensitivity analysis with *time‐invariant covariates with stable effects* were mostly in line with the main analysis. All significant parameter estimates signficant after applying *k‐*FWER corrections were also significant after adjusting for baseline covariates; beta weights were also approximately equal in size. Testing the assumption of stable effects of baseline covariates, we found that the model allowing *time‐invariant covariates to have time‐varying effects* fit better than the model with covariates forced to have stable effects (females: *∆BIC* = 556.98; males: *∆BIC* = 365.97). Thus, this assumption does not hold for the variables included in our model. However, as in the model that assumes stable effects, including covariates did not substantially affect our within‐person estimates. Including baseline covariates affected very few parameters (mostly becoming nonsignificant). With one exception, these parameters were also not significant after correcting for multiple comparisons. The only substantive difference between the model including baseline factors as time‐invariant covariates with time‐varying effects compared to other models was that, in the model for male children, a cross‐lagged effect of externalising on maternal mental health problems was observed from age 3 to age 5, while this effect was previously observed from age 5 to age 7. Overall, the sensitivity analysis results suggest that the ALT‐SR generally controls well for the effects of time‐invariant covariates.

## Discussion

We tested a transactional model of within‐family relations between maternal, paternal and child mental health over child and adolescent development, using an ALT‐SR to disaggregate between‐ from within‐person/family effects. Overall, results supported a transactional model, with some caveats and restrictions. Specifically, maternal distress was associated with increased internalising problems in boys and girls, but with increased externalising problems in girls only. For boys, paternal psychological distress was only associated with increased internalising and externalising problems during early adolescence. For girls, paternal psychological distress was associated with lower internalising and externalising problems during early childhood. Parent‐to‐child effects were not robust to corrections for multiple comparisons. Regarding child‐to‐parent effects, internalising problems in boys but externalising problems in girls were associated with later maternal psychological distress. Child‐to‐father effects were limited to middle childhood and showed the reverse pattern, with internalising problems in girls but externalising problems in boys being associated with subsequent paternal mental health difficulties. Both child‐to‐parent and parent‐to‐child effects showed developmentally dynamic associations, highlighting the importance of ensuring investigations encompass a wide developmental span.

Our finding that maternal psychological distress was associated with internalising problems in both boys and girls but with externalising problems in girls only contrasts with previous research finding no gender differences (Xerxa et al., 2021) or that maternal depression is predominantly associated with increases in boys’ externalising problems (e.g. Choe et al., 2014). However, our results on the effects of maternal psychological distress on girls’ mental health are in line with previous research (Tyrell et al., 2019). One potential mechanism for these mother‐to‐child effects could be that increases in maternal mental health problems can lead to a coercive cycle of maladaptive parent–child interactions that increase child behavioural problems (Patterson, 2002). Maternal mental health problems could also affect mother–child relationships to result in insecure attachment, which is known to be related to child mental health, particularly internalising problems (Spruit et al., 2019). However, effects of maternal mental health were only observed during limited developmental periods, were of small effect size and were no longer statistically significant after correcting for multiple comparisons.

Father‐to‐child effects also differed based on the child’s gender, were of small effect and were not robust to corrections for multiple comparisons. For boys, paternal psychological distress was associated with increased internalising and externalising problems during early adolescence, suggesting that this period might be particularly crucial in father–son transactions. For girls, paternal psychological distress in early childhood was associated with decreased internalising and externalising problems. One potential explanation may be that growing up with a father with mental health difficulties might make daughters more inhibited, decreasing their likelihood of developing externalising problems. However, this would imply that such girls would develop more internalising problems, which we did not observe here. Indeed, this finding was unexpected considering previous evidence that paternal mental health problems lead to increased offspring mental health difficulties in childhood (Xerxa et al., 2021) and adolescence (Tyrell et al., 2019). Further research is needed to clarify the nature of this association.

Supporting a transactional model of child development, alongside the (albeit weak) parent‐to‐child effects we found evidence for child‐to‐parent effects. Parenting stress may contribute to the association between child and parental mental health problems, as it appears to be: (a) higher among parents of children with emotional and behavioural problems (Barroso, Mendez, Graziano, & Bagner, 2018); and (b) linked to parental mental health problems such as depression and anxiety (Farmer & Lee, 2011). Mothers of children with internalising or externalising problems may feel that parenting demands outstrip their resources, leading to feelings of incompetence and lack of control, resulting in symptoms of depression. As with the relation between parental and child mental health, the directionality between parenting stress, children’s behaviours and parental mental health is unclear: evidence points to reciprocal relations, such that parenting stress may also help explain parent‐to‐child effects (e.g. Stone, Mares, Otten, Engels, & Janssens, 2016). Interestingly, Stone et al. (2016) found that parenting stress was more strongly associated with externalising problems in boys than girls; this contrasts with our findings, which showed that maternal psychological distress was associated with externalising problems in girls, but with internalising problems in boys. Given previous consistent evidence of elevated externalising problems in boys and elevated internalising problems in girls (e.g. Eaton et al., 2012), our findings suggest that mothers are more adversely affected by children’s mental health difficulties when they are less gender‐typical and, by extension, more unexpected. Indeed, prior research shows that gender‐atypical behaviour is associated with increases in negative parent–child interactions (Alanko et al., 2011), which are in turn closely linked to parental mental health problems (Lovejoy, Graczyk, O’Hare, & Neuman, 2000). Equally, mothers may succeed in identifying gender‐typical mental health issues but attribute gender‐atypical issues to their child being deliberately difficult. By contrast, fathers were more likely to be affected by gender‐typical child mental health issues. To date, few studies have investigated gender differences in parent‐to‐child mental health relations, and thus, our results require replication, ideally in studies using multi‐informant reports.

Finally, our results suggest developmentally dynamic reciprocal relations between parental mental health difficulties. In early childhood, parents showed possible compensatory effects, with negative associations between each parent’s mental health problems. This was unexpected, as previous research indicates within‐couple concordance in parents’ mental health trajectories (e.g. Hughes et al., 2020). However, our findings show a similar concordance in parental psychological distress in middle childhood and adolescence. To our knowledge, this is the first study to track the interrelations between parents’ mental health from early childhood to adolescence. One potential reason for the observed developmental differences could be that young children are highly dependent on at least one parent, who consequently has to step in if the other parent is struggling with their well‐being. Such compensatory effects may protect that parent from developing mental health problems during early childhood. However, this early compensatory behaviour may not be sustainable in the long term, leading to greater between‐parent concordance in mental health difficulties later on. These effects may only become apparent when within‐ and between‐family effects are disaggregated, underscoring the importance of doing so in future research.

A key strength of this study is the simultaneous investigation of reciprocal relations in paternal, maternal and child mental health over childhood and adolescence in a large nationally representative sample. Crucially, we used a statistical design that offers more direct insights into within‐family dynamics. Indeed, unlike the methods used in most previous studies, ALT‐SRs can disaggregate stable between‐family effects from within‐family effects that are referenced in transactional theories (Hamaker et al., 2015). This was also supported by our sensitivity analyses that included a range of baseline covariates. Arguably, our results therefore offer some of the most robust evidence on within‐family relations of mental health problems to date. It is important to note that longitudinal studies that control for stability effects tend to yield smaller results. Thus, effects such as those identified in the current study that may be considered trivial following traditional effect size classifications may still be meaningful, especially if they accumulate over time (Adachi & Willoughby, 2014).

Several study limitations also deserve note. ALT‐SRs control for stable between‐person differences in mental health problems, but are vulnerable to time‐varying confounds. For instance, unmeasured life events occurring at a specific time point (e.g. death of a family member) could have influenced the observed within‐family relations by affecting a child’s mental health symptoms independently from prior parental mental health problems. Further, shared‐rater bias is a potential issue as children’s problems were primarily rated by their mothers (Xerxa et al., 2021), which may also explain some of the observed differences in mother–child effects compared to father–child effects. However, the current study focused on longitudinal associations that spanned at least two years, thus later reports are likely less confounded by maternal mental health problems than concurrent reports.

## Conclusion

Supporting a transactional model of child development, our results demonstrate child‐to‐parent, parent‐to‐child and parent‐to‐parent effects (with child‐to‐mother effects being especially consistent). These findings indicate that the whole family system should be included in intervention efforts. Family‐focused interventions such as family system therapy may prove effective in reducing both parental and child mental health difficulties. Future studies are needed to investigate potential mediating factors in the transactions between parental and child mental health such as coercive parenting and parent–child relationship quality, as well as moderating factors such as family structure or socio‐economic status.

## Supporting information


**Table S1**. Sample demographic information at baseline.
**Table S2**. Descriptive Statistics.
**Table S3**. Standardised autoregressive and cross‐lagged parameters for the ALT‐SR for boys.
**Table S4**. Latent Growth Curve Parameters.
**Table S5**. Residual correlations.
**Table S6**. Standardised autoregressive and cross‐lagged parameters for the ALT‐SR for girls.
**Appendix S1**. Kessler (K6) Scale.
