# Supplementary material for: Within‐family relations of mental health problems across childhood and adolescence
Source: J Child Psychol Psychiatry. 2022 Jan 24;63(11):1288–96. doi: 10.1111/jcpp.13572 (PMC9787478; doi:10.1111/jcpp.13572)
Supplement: Supplementary file 1 — Table S1. Sample demographic information at baseline. Table S2. Descriptive Statistics. Table S3. Standardised autoregressive and cross‐lagged parameters for the ALT‐SR for boys. Table S4. Latent Growth Curve Parameters. Table S5. Residual correlations. Table S6. Standardised autoregressive and cross‐lagged parameters for the ALT‐SR for girls. Appendix S1. Kessler (K6) Scale. [file JCPP-63-1288-s001.docx]

**Supporting Information**

| **Table S1.** Sample demographic information at baseline | | | |
| --- | --- | --- | --- |
| **Variable** | **Category** | **%** | **N** |
| **Child Sex** | Female | 50.03 | 5376 |
|  | Male | 49.97 | 5370 |
| **Child Ethnicity** | White | 81.70 | 8466 |
|  | Other Ethnicity | 18.30 | 1896 |
| **Maternal Ethnicity** | White | 82.99 | 8589 |
|  | Other Ethnicity | 17.01 | 1761 |
| **Paternal Ethnicity** | White | 84.91 | 6791 |
|  | Other Ethnicity | 15.09 | 1201 |
| **Maternal Academic Qualification** | Higher Degree | 4.17 | 432 |
|  | First Degree | 15.83 | 1639 |
|  | Diplomas in Higher Education | 9.45 | 979 |
|  | A/AS/S Levels | 10.11 | 1046 |
|  | O level/GCSE Grades A-C | 32.35 | 3358 |
|  | GCSE Grades A-C | 9.30 | 962 |
|  | Other Academic Qualification | 2.94 | 304 |
|  | None of these Qualifications | 15.84 | 1639 |
| **Paternal Academic Qualification** | Higher Degree | 6.35 | 508 |
|  | First Degree | 16.74 | 1339 |
|  | Diplomas in Higher Education | 9.53 | 762 |
|  | A/AS/S Levels | 8.04 | 643 |
|  | O level/GCSE Grades A-C | 30.25 | 2419 |
|  | GCSE Grades A-C | 9.22 | 737 |
|  | Other Academic Qualification | 2.70 | 216 |
|  | None of these Qualifications | 17.16 | 1373 |
| **Deprivation** | Most Deprived Decile | 13.91 | 1375 |
|  | 10 - <20% | 12.25 | 1211 |
|  | 20 - <30% | 11.17 | 1104 |
|  | 30 - <40% | 9.67 | 956 |
|  | 40 - <50% | 9.24 | 913 |
|  | 50 - <60% | 8.92 | 882 |
|  | 60 - <70% | 8.01 | 792 |
|  | 70 - <80% | 8.00 | 791 |
|  | 80 - <90% | 9.13 | 903 |
|  | Least Deprived Decile | 9.70 | 959 |
|  |  | ***Mean*** | ***SD*** |
| **Child Age at Data Collection** | Wave 2 | 3.13 | 0.19 |
|  | Wave 3 | 5.21 | 0.24 |
|  | Wave 4 | 7.23 | 0.24 |
|  | Wave 5 | 10.66 | 0.48 |
|  | Wave 6 | 13.76 | 0.45 |
|  | Wave 7 | 16.69 | 0.47 |
| *Note.* These are based on the sample of participants with data up to the age 17 wave | | | |

| **Table S2.** Descriptive Statistics | | | | | | | | | |
| --- | --- | --- | --- | --- | --- | --- | --- | --- | --- |
|  | ***N*** | ***% NA*** | ***μ*** | ***SD*** | ***Min*** | ***Max*** | ***Skew*** | ***Kurtosis*** | ***ω*** |
| Age 3 internalising problems | 9267 | .14 | 2.84 | 2.49 | 0 | 18 | 1.27 | 1.96 | .77 |
| Age 5 internalising problems | 9784 | .09 | 2.48 | 2.52 | 0 | 18 | 1.57 | 3.17 | .79 |
| Age 7 internalising problems | 9593 | .11 | 2.68 | 2.74 | 0 | 18 | 1.48 | 2.56 | .81 |
| Age 11 internalising problems | 9708 | .10 | 3.17 | 3.14 | 0 | 19 | 1.42 | 2.19 | .84 |
| Age 14 internalising problems | 9579 | .11 | 3.73 | 3.40 | 0 | 19 | 1.25 | 1.49 | .86 |
| Age 17 internalising problems | 9363 | .13 | 3.81 | 3.48 | 0 | 18 | 1.19 | 1.12 | .87 |
| Age 3 externalising problems | 9280 | .14 | 6.54 | 3.75 | 0 | 20 | 0.58 | 0.01 | .79 |
| Age 5 externalising problems | 9761 | .09 | 4.63 | 3.36 | 0 | 20 | 0.88 | 0.61 | .83 |
| Age 7 externalising problems | 9585 | .11 | 4.57 | 3.52 | 0 | 19 | 0.88 | 0.46 | .86 |
| Age 11 externalising problems | 9691 | .10 | 4.33 | 3.51 | 0 | 20 | 1.02 | 0.88 | .88 |
| Age 14 externalising problems | 9572 | .11 | 4.27 | 3.50 | 0 | 20 | 1.10 | 1.24 | .90 |
| Age 17 externalising problems | 9363 | .13 | 3.67 | 3.33 | 0 | 20 | 1.25 | 1.68 | .90 |
| Age 3 maternal psychological distress | 8473 | .21 | 3.16 | 3.59 | 0 | 24 | 1.91 | 4.46 | NA |
| Age 5 maternal psychological distress | 9404 | .12 | 3.08 | 3.67 | 0 | 24 | 1.92 | 4.52 | NA |
| Age 7 maternal psychological distress | 9217 | .14 | 3.04 | 3.73 | 0 | 24 | 1.97 | 4.70 | NA |
| Age 11 maternal psychological distress | 6819 | .37 | 3.78 | 3.98 | 0 | 24 | 1.68 | 3.37 | NA |
| Age 14 maternal psychological distress | 7049 | .34 | 4.07 | 3.97 | 0 | 24 | 1.56 | 2.91 | NA |
| Age 17 maternal psychological distress | 7286 | .32 | 4.21 | 4.40 | 0 | 24 | 1.46 | 2.05 | NA |
| Age 3 paternal psychological distress | 6569 | .39 | 2.88 | 3.04 | 0 | 24 | 1.76 | 4.51 | NA |
| Age 5 paternal psychological distress | 6850 | .36 | 2.93 | 3.24 | 0 | 24 | 1.89 | 4.97 | NA |
| Age 7 paternal psychological distress | 6369 | .41 | 2.94 | 3.34 | 0 | 24 | 1.85 | 4.39 | NA |
| Age 11 paternal psychological distress | 5922 | .45 | 3.52 | 3.81 | 0 | 24 | 1.80 | 4.14 | NA |
| Age 14 paternal psychological distress | 5423 | .50 | 3.63 | 3.59 | 0 | 24 | 1.71 | 4.06 | NA |
| Age 17 paternal psychological distress | 4522 | .58 | 3.55 | 3.83 | 0 | 24 | 1.69 | 3.63 | NA |
| *Note. % NA* = percent missing at each data collection wave; *μ* = mean; *ω =* McDonalds Omega using polychoric correlations (internal consistency). Ages correspond to children’s median ages at data collection. Internal consistency values were not available for the parental mental health measures as the sum-scores provided by the Millennium Cohort Study were used in the current study. Previous studies using the Kessler (K6) scale have indicated that it has good reliability (Flouri et al., 2019). | | | | | | | | | |

**Appendix S1. Kessler (K6) Scale – items:**

1. During the last 30 days, about how often did you feel so depressed that nothing could cheer you up?
2. During the last 30 days, about how often did you feel hopeless?
3. During the last 30 days, about how often did you feel restless or fidgety?
4. During the last 30 days, about how often did you feel that everything was an effort?
5. During the last 30 days, about how often did you feel worthless?
6. During the last 30 days, about how often did you feel nervous?

| **Table S3.** Standardised autoregressive and cross-lagged parameters for the ALT-SR for boys | | | | |
| --- | --- | --- | --- | --- |
| **Parameter** | ***Estimate*** | ***SE*** | ***p*** | ***q*** |
| Age 17 internalising on age 14 internalising | 0.540 | 0.028 | <.001* | <.001** |
| Age 17 internalising on age 14 externalising | 0.099 | 0.035 | .005* | .048** |
| Age 17 internalising on age 14 maternal psychological distress | 0.027 | 0.029 | .352 | .999 |
| Age 17 internalising on age 14 paternal psychological distress | -0.004 | 0.047 | .938 | .999 |
| Age 17 externalising on age 14 internalising | 0.102 | 0.034 | .003* | .031** |
| Age 17 externalising on age 14 externalising | 0.507 | 0.033 | <.001* | <.001** |
| Age 17 externalising on age 14 maternal psychological distress | 0.002 | 0.031 | .943 | .999 |
| Age 17 externalising on age 14 paternal psychological distress | 0.015 | 0.036 | .684 | .999 |
| Age 17 maternal psychological distress on age 14 internalising | 0.095 | 0.037 | .011* | .099 |
| Age 17 maternal psychological distress on age 14 externalising | 0.031 | 0.037 | .403 | .999 |
| Age 17 maternal psychological distress on age 14 maternal psychological distress | 0.416 | 0.046 | <.001* | <.001** |
| Age 17 maternal psychological distress on age 14 paternal psychological distress | 0.066 | 0.051 | .191 | .999 |
| Age 17 paternal psychological distress on age 14 internalising | 0.078 | 0.043 | .073 | .547 |
| Age 17 paternal psychological distress on age 14 externalising | 0.041 | 0.040 | .302 | .999 |
| Age 17 paternal psychological distress on age 14 maternal psychological distress | 0.047 | 0.044 | .278 | .999 |
| Age 17 paternal psychological distress on age 14 paternal psychological distress | 0.365 | 0.062 | <.001* | <.001** |
| Age 14 internalising on age 14 internalising | 0.427 | 0.038 | <.001* | <.001** |
| Age 14 internalising on age 11 externalising | 0.174 | 0.036 | <.001* | <.001** |
| Age 14 internalising on age 11 maternal psychological distress | 0.013 | 0.034 | .712 | .999 |
| Age 14 internalising on age 11 paternal psychological distress | 0.089 | 0.033 | .008* | .073 |
| Age 14 externalising on age 11 internalising | 0.096 | 0.031 | .002* | .021** |
| Age 14 externalising on age 11 externalising | 0.507 | 0.031 | <.001* | <.001** |
| Age 14 externalising on age 11 maternal psychological distress | 0.030 | 0.029 | .301 | .999 |
| Age 14 externalising on age 11 paternal psychological distress | 0.062 | 0.028 | .029* | .232 |
| Age 14 maternal psychological distress on age 11 internalising | 0.128 | 0.044 | .004* | .039** |
| Age 14 maternal psychological distress on age 14 externalising | 0.061 | 0.045 | .181 | .999 |
| Age 14 maternal psychological distress on age 14 maternal psychological distress | -0.008 | 0.042 | .840 | .999 |
| Age 14 maternal psychological distress on age 14 paternal psychological distress | 0.355 | 0.046 | <.001* | <.001** |
| Age 14 paternal psychological distress on age 14 internalising | -0.032 | 0.072 | .660 | .999 |
| Age 14 paternal psychological distress on age 14 externalising | 0.061 | 0.060 | .311 | .999 |
| Age 14 paternal psychological distress on age 14 maternal psychological distress | 0.497 | 0.060 | <.001* | <.001** |
| Age 14 paternal psychological distress on age 14 paternal psychological distress | 0.029 | 0.056 | .605 | .999 |
| Age 11 internalising on age 7 internalising | 0.307 | 0.034 | <.001* | <.001** |
| Age 11 internalising on age 7 externalising | 0.199 | 0.035 | <.001* | <.001** |
| Age 11 internalising on age 7 maternal psychological distress | 0.021 | 0.030 | .479 | .999 |
| Age 11 internalising on age 7 paternal psychological distress | 0.006 | 0.033 | .858 | .999 |
| Age 11 externalising on age 7 internalising | 0.055 | 0.031 | .080 | .573 |
| Age 11 externalising on age 7 externalising | 0.440 | 0.035 | <.001* | <.001** |
| Age 11 externalising on age 7 maternal psychological distress | 0.054 | 0.036 | .130 | .888 |
| Age 11 externalising on age 7 paternal psychological distress | -0.007 | 0.034 | .838 | .999 |
| Age 11 maternal psychological distress on age 7 internalising | 0.089 | 0.039 | .022* | .183 |
| Age 11 maternal psychological distress on age 7 externalising | 0.061 | 0.045 | .177 | .999 |
| Age 11 maternal psychological distress on age 7 maternal psychological distress | -0.135 | 0.045 | .003* | .031** |
| Age 11 maternal psychological distress on age 7 paternal psychological distress | 0.282 | 0.049 | <.001* | <.001** |
| Age 11 paternal psychological distress on age 7 internalising | 0.017 | 0.037 | .650 | .999 |
| Age 11 paternal psychological distress on age 7 externalising | 0.088 | 0.042 | .034* | .266 |
| Age 11 paternal psychological distress on age 7 maternal psychological distress | 0.344 | 0.050 | <.001* | <.001** |
| Age 11 paternal psychological distress on age 7 paternal psychological distress | -0.071 | 0.053 | .178 | .999 |
| Age 7 internalising on age 5 internalising | 0.284 | 0.038 | <.001* | <.001** |
| Age 7 internalising on age 5 externalising | 0.138 | 0.032 | <.001* | <.001** |
| Age 7 internalising on age 5 maternal psychological distress | 0.091 | 0.033 | .007* | .065 |
| Age 7 internalising on age 5 paternal psychological distress | -0.016 | 0.031 | .611 | .999 |
| Age 7 externalising on age 5 internalising | 0.008 | 0.029 | .779 | .999 |
| Age 7 externalising on age 5 externalising | 0.489 | 0.033 | <.001* | <.001** |
| Age 7 externalising on age 5 maternal psychological distress | 0.033 | 0.031 | .292 | .999 |
| Age 7 externalising on age 5 paternal psychological distress | -0.014 | 0.029 | .625 | .999 |
| Age 7 maternal psychological distress on age 5 internalising | 0.026 | 0.039 | .500 | .999 |
| Age 7 maternal psychological distress on age 5 externalising | 0.111 | 0.037 | .003* | .031** |
| Age 7 maternal psychological distress on age 5 maternal psychological distress | 0.222 | 0.043 | <.001* | <.001** |
| Age 7 maternal psychological distress on age 5 paternal psychological distress | -0.100 | 0.042 | .017* | .145 |
| Age 7 paternal psychological distress on age 5 internalising | -0.059 | 0.040 | .140 | .933 |
| Age 7 paternal psychological distress on age 5 externalising | 0.068 | 0.038 | .074 | .547 |
| Age 7 paternal psychological distress on age 5 maternal psychological distress | -0.084 | 0.038 | .028* | .229 |
| Age 7 paternal psychological distress on age 5 paternal psychological distress | 0.228 | 0.050 | <.001* | <.001** |
| Age 5 internalising on age 3 internalising | 0.108 | 0.058 | .061 | .468 |
| Age 5 internalising on age 3 externalising | 0.016 | 0.043 | .713 | .999 |
| Age 5 internalising on age 3 maternal psychological distress | 0.021 | 0.052 | .691 | .999 |
| Age 5 internalising on age 3 paternal psychological distress | -0.102 | 0.065 | .119 | .833 |
| Age 5 externalising on age 3 internalising | -0.117 | 0.049 | .016* | .139 |
| Age 5 externalising on age 3 externalising | 0.274 | 0.039 | <.001* | <.001** |
| Age 5 externalising on age 3 maternal psychological distress | 0.030 | 0.044 | .500 | .999 |
| Age 5 externalising on age 3 paternal psychological distress | -0.022 | 0.043 | .610 | .999 |
| Age 5 maternal psychological distress on age 3 internalising | -0.024 | 0.039 | .543 | .999 |
| Age 5 maternal psychological distress on age 3 externalising | 0.049 | 0.035 | .158 | .999 |
| Age 5 maternal psychological distress on age 3 maternal psychological distress | 0.153 | 0.052 | .003* | .031** |
| Age 5 maternal psychological distress on age 3 paternal psychological distress | -0.193 | 0.044 | <.001* | <.001** |
| Age 5 paternal psychological distress on age 3 internalising | 0.005 | 0.056 | .921 | .999 |
| Age 5 paternal psychological distress on age 3 externalising | -0.014 | 0.043 | .751 | .999 |
| Age 5 paternal psychological distress on age 3 maternal psychological distress | -0.181 | 0.049 | <.001* | <.001** |
| Age 5 paternal psychological distress on age 3 paternal psychological distress | 0.146 | 0.057 | .011* | .099 |
| *Note.* *significant at *p*<.05 (unadjusted). **significant at *q*<.05 (adjusted). Ages correspond to children’s median ages at data collection. | | | | |

| **Table S4.** Latent Growth Curve Parameters | | | | | |
| --- | --- | --- | --- | --- | --- |
| **(a) ALT-SR for boys** |  | ***B*** | ***SE*** | ***p*** | ***q*** |
| Internalising Problems | Intercept | 2.756 | 0.067 | <.001* | <.001** |
|  | Linear Slope | 0.923 | 0.269 | .001* | .001** |
|  | Quadratic Slope | 0.016 | 0.228 | .944 | .944 |
| Externalising Problems | Intercept | 6.548 | 0.112 | <.001* | <.001** |
|  | Linear Slope | -3.412 | 0.295 | <.001* | <.001** |
|  | Quadratic Slope | 1.383 | 0.267 | <.001* | <.001** |
| Maternal Psychological Distress | Intercept | 3.259 | 0.081 | <.001* | <.001** |
|  | Slope | 1.431 | 0.117 | <.001* | <.001** |
| Paternal Psychological Distress | Intercept | 3.194 | 0.094 | <.001* | <.001** |
|  | Slope | 1.169 | 0.127 | <.001* | <.001** |
|  |  |  |  |  |  |
| **(b) ALT-SR for girls** |  | ***B*** | ***SE*** | ***p*** | ***q*** |
| Internalising Problems | Intercept | 2.563 | 0.071 | <.001* | <.001** |
|  | Linear Slope | -0.181 | 0.255 | .479 | .479 |
|  | Quadratic Slope | 2.05 | 0.274 | <.001* | <.001** |
| Externalising Problems | Intercept | 5.546 | 0.105 | <.001* | <.001** |
|  | Linear Slope | -4.555 | 0.303 | <.001* | <.001** |
|  | Quadratic Slope | 2.488 | 0.27 | <.001* | <.001** |
| Maternal Psychological Distress | Intercept | 3.01 | 0.091 | <.001* | <.001** |
|  | Slope | 1.428 | 0.114 | <.001* | <.001** |
| Paternal Psychological Distress | Intercept | 2.921 | 0.086 | <.001* | <.001** |
|  | Slope | 1.157 | 0.112 | <.001* | <.001** |
| *Note.* *significant at *p*<.05 (unadjusted). **significant at *q*<.05 (adjusted). | | | | | |

| **Table S5.** Residual correlations | | | | | | | | | | | | |
| --- | --- | --- | --- | --- | --- | --- | --- | --- | --- | --- | --- | --- |
| **(a) ALT-SR for boys** | | | | | | | | | | | | |
|  | **Age 3** | | | | **Age 5** | | | | **Age 7** | | | |
|  | 1. | 2. | 3. | 4. | 1. | 2. | 3. | 4. | 1. | 2. | 3. | 4. |
| 1. Internalising problems | - |  |  |  | - |  |  |  | - |  |  |  |
| 2. Externalising problems | .19** | - |  |  | .27** | - |  |  | .32** | - |  |  |
| 3. Maternal psychological distress | .05 | 0.15** | - |  | .18** | .17** | - |  | .25** | .23** | - |  |
| 4. Paternal psychological distress | -.05 | -0.05 | -0.10 | - | -.08 | .05 | -.06 | - | .04 | .05 | .13** | - |
|  | **Age 11** | | | | **Age 14** | | | | **Age 17** | | | |
|  | 1. | 2. | 3. | 4. | 1. | 2. | 3. | 4. | 1. | 2. | 3. | 4. |
| 1. Internalising problems | - |  |  |  | - |  |  |  | - |  |  |  |
| 2. Externalising problems | .40** | - |  |  | .36** | - |  |  | .36** | - |  |  |
| 3. Maternal psychological distress | .15** | 0.16** | - |  | .13** | .03 | - |  | .15** | .15** | - |  |
| 4. Paternal psychological distress | .17** | 0.16** | 0.02 | - | -.02 | .05 | .13** | - | .08* | .10** | .20** | - |
| **(b) ALT-SR for girls** | | | | | | | | | | | | |
|  | **Age 3** | | | | **Age 5** | | | | **Age 7** | | | |
|  | 1. | 2. | 3. | 4. | 1. | 2. | 3. | 4. | 1. | 2. | 3. | 4. |
| 1. Internalising problems | - |  |  |  | - |  |  |  | - |  |  |  |
| 2. Externalising problems | .25** | - |  |  | .25** | - |  |  | .22** | - |  |  |
| 3. Maternal psychological distress | .04 | 0.14** | - |  | .09* | .18** | - |  | .14** | .10** | - |  |
| 4. Paternal psychological distress | -.17** | -0.11* | -0.10 | - | -.10* | -.01 | .00 | - | .00 | .04 | .22** | - |
|  | **Age 11** | | | | **Age 14** | | | | **Age 17** | | | |
|  | 1. | 2. | 3. | 4. | 1. | 2. | 3. | 4. | 1. | 2. | 3. | 4. |
| 1. Internalising problems | - |  |  |  | - |  |  |  | - |  |  |  |
| 2. Externalising problems | .39** | - |  |  | .35** | - |  |  | .38** | - |  |  |
| 3. Maternal psychological distress | .16** | 0.22** | - |  | .19** | .18** | - |  | .17** | .14** | - |  |
| 4. Paternal psychological distress | .17** | 0.14** | -0.01 | - | .10* | .09* | .10** | - | .11** | .12** | .17** | - |
| *Note.* *significant at *p*<.05 (unadjusted). **significant at *q*<.05 (adjusted). Ages correspond to children’s median ages at data collection. | | | | | | | | | | | | |

| **Table S6.** Standardised autoregressive and cross-lagged parameters for the ALT-SR for girls | | | | |
| --- | --- | --- | --- | --- |
| **Parameter** | ***Est.*** | ***SE*** | ***p*** | ***q*** |
| Age 17 internalising on age 14 internalising | 0.483 | 0.023 | <.001* | <.001** |
| Age 17 internalising on age 14 externalising | 0.086 | 0.030 | .004* | .039** |
| Age 17 internalising on age 14 maternal psychological distress | 0.019 | 0.033 | .575 | .999 |
| Age 17 internalising on age 14 paternal psychological distress | 0.063 | 0.036 | .078 | .546 |
| Age 17 externalising on age 14 internalising | 0.162 | 0.033 | <.001* | <.001** |
| Age 17 externalising on age 14 externalising | 0.374 | 0.038 | <.001* | <.001** |
| Age 17 externalising on age 14 maternal psychological distress | -0.017 | 0.027 | .533 | .999 |
| Age 17 externalising on age 14 paternal psychological distress | 0.029 | 0.037 | .429 | .999 |
| Age 17 maternal psychological distress on age 14 internalising | 0.069 | 0.032 | .034* | .278 |
| Age 17 maternal psychological distress on age 14 externalising | 0.113 | 0.036 | .002* | .020** |
| Age 17 maternal psychological distress on age 14 maternal psychological distress | 0.433 | 0.036 | <.001* | <.001** |
| Age 17 maternal psychological distress on age 14 paternal psychological distress | 0.106 | 0.041 | .010* | .095 |
| Age 17 paternal psychological distress on age 14 internalising | 0.038 | 0.033 | .245 | .999 |
| Age 17 paternal psychological distress on age 14 externalising | -0.018 | 0.045 | .696 | .999 |
| Age 17 paternal psychological distress on age 14 maternal psychological distress | -0.017 | 0.041 | .678 | .999 |
| Age 17 paternal psychological distress on age 14 paternal psychological distress | 0.467 | 0.048 | <.001* | <.001** |
| Age 14 internalising on age 14 internalising | 0.393 | 0.026 | <.001* | <.001** |
| Age 14 internalising on age 11 externalising | 0.165 | 0.028 | <.001* | <.001** |
| Age 14 internalising on age 11 maternal psychological distress | 0.009 | 0.030 | .767 | .999 |
| Age 14 internalising on age 11 paternal psychological distress | 0.032 | 0.027 | .234 | .999 |
| Age 14 externalising on age 11 internalising | 0.045 | 0.034 | .192 | .999 |
| Age 14 externalising on age 11 externalising | 0.397 | 0.040 | <.001* | <.001** |
| Age 14 externalising on age 11 maternal psychological distress | 0.054 | 0.035 | .125 | .729 |
| Age 14 externalising on age 11 paternal psychological distress | 0.053 | 0.028 | .053 | .397 |
| Age 14 maternal psychological distress on age 11 internalising | 0.020 | 0.034 | .560 | .999 |
| Age 14 maternal psychological distress on age 14 externalising | 0.093 | 0.043 | .031* | .258 |
| Age 14 maternal psychological distress on age 14 maternal psychological distress | 0.084 | 0.045 | .062 | .455 |
| Age 14 maternal psychological distress on age 14 paternal psychological distress | 0.345 | 0.047 | <.001* | <.001** |
| Age 14 paternal psychological distress on age 14 internalising | 0.017 | 0.033 | .604 | .999 |
| Age 14 paternal psychological distress on age 14 externalising | -0.016 | 0.042 | .700 | .999 |
| Age 14 paternal psychological distress on age 14 maternal psychological distress | 0.457 | 0.046 | <.001* | <.001** |
| Age 14 paternal psychological distress on age 14 paternal psychological distress | 0.077 | 0.045 | .087 | .595 |
| Age 11 internalising on age 7 internalising | 0.287 | 0.025 | <.001* | <.001** |
| Age 11 internalising on age 7 externalising | 0.132 | 0.030 | <.001* | <.001** |
| Age 11 internalising on age 7 maternal psychological distress | 0.059 | 0.028 | .037* | .296 |
| Age 11 internalising on age 7 paternal psychological distress | -0.001 | 0.033 | .966 | .999 |
| Age 11 externalising on age 7 internalising | 0.065 | 0.026 | .011* | .103 |
| Age 11 externalising on age 7 externalising | 0.433 | 0.031 | <.001* | <.001** |
| Age 11 externalising on age 7 maternal psychological distress | 0.074 | 0.032 | .022* | .191 |
| Age 11 externalising on age 7 paternal psychological distress | -0.021 | 0.034 | .526 | .999 |
| Age 11 maternal psychological distress on age 7 internalising | 0.037 | 0.037 | .310 | .999 |
| Age 11 maternal psychological distress on age 7 externalising | 0.053 | 0.034 | .121 | .726 |
| Age 11 maternal psychological distress on age 7 maternal psychological distress | -0.090 | 0.046 | .048* | .376 |
| Age 11 maternal psychological distress on age 7 paternal psychological distress | 0.357 | 0.044 | <.001* | <.001** |
| Age 11 paternal psychological distress on age 7 internalising | 0.138 | 0.038 | <.001* | <.001** |
| Age 11 paternal psychological distress on age 7 externalising | 0.060 | 0.038 | .111 | .684 |
| Age 11 paternal psychological distress on age 7 maternal psychological distress | 0.290 | 0.048 | <.001* | <.001** |
| Age 11 paternal psychological distress on age 7 paternal psychological distress | -0.080 | 0.049 | .102 | .663 |
| Age 7 internalising on age 5 internalising | 0.230 | 0.035 | <.001* | <.001** |
| Age 7 internalising on age 5 externalising | 0.099 | 0.032 | .002* | .020** |
| Age 7 internalising on age 5 maternal psychological distress | 0.014 | 0.032 | .660 | .999 |
| Age 7 internalising on age 5 paternal psychological distress | -0.058 | 0.034 | .090 | .600 |
| Age 7 externalising on age 5 internalising | -0.036 | 0.027 | .175 | .962 |
| Age 7 externalising on age 5 externalising | 0.524 | 0.030 | <.001* | <.001** |
| Age 7 externalising on age 5 maternal psychological distress | 0.048 | 0.024 | .048* | .376 |
| Age 7 externalising on age 5 paternal psychological distress | -0.072 | 0.030 | .018* | .162 |
| Age 7 maternal psychological distress on age 5 internalising | 0.022 | 0.037 | .554 | .999 |
| Age 7 maternal psychological distress on age 5 externalising | 0.124 | 0.030 | <.001* | <.001** |
| Age 7 maternal psychological distress on age 5 maternal psychological distress | 0.290 | 0.043 | <.001* | <.001** |
| Age 7 maternal psychological distress on age 5 paternal psychological distress | 0.021 | 0.040 | .594 | .999 |
| Age 7 paternal psychological distress on age 5 internalising | -0.069 | 0.038 | .067 | .480 |
| Age 7 paternal psychological distress on age 5 externalising | 0.051 | 0.033 | .126 | .729 |
| Age 7 paternal psychological distress on age 5 maternal psychological distress | 0.022 | 0.045 | .628 | .999 |
| Age 7 paternal psychological distress on age 5 paternal psychological distress | 0.251 | 0.051 | <.001* | <.001** |
| Age 5 internalising on age 3 internalising | 0.100 | 0.043 | .020* | .177 |
| Age 5 internalising on age 3 externalising | 0.033 | 0.030 | .265 | .999 |
| Age 5 internalising on age 3 maternal psychological distress | -0.073 | 0.045 | .104 | .663 |
| Age 5 internalising on age 3 paternal psychological distress | -0.111 | 0.049 | .023* | .196 |
| Age 5 externalising on age 3 internalising | -0.026 | 0.036 | .457 | .999 |
| Age 5 externalising on age 3 externalising | 0.305 | 0.032 | <.001* | <.001** |
| Age 5 externalising on age 3 maternal psychological distress | -0.006 | 0.042 | .895 | .999 |
| Age 5 externalising on age 3 paternal psychological distress | -0.013 | 0.043 | .772 | .999 |
| Age 5 maternal psychological distress on age 3 internalising | -0.033 | 0.042 | .429 | .999 |
| Age 5 maternal psychological distress on age 3 externalising | 0.014 | 0.035 | .697 | .999 |
| Age 5 maternal psychological distress on age 3 maternal psychological distress | 0.164 | 0.060 | .006* | .058 |
| Age 5 maternal psychological distress on age 3 paternal psychological distress | -0.166 | 0.047 | <.001* | <.001** |
| Age 5 paternal psychological distress on age 3 internalising | -0.156 | 0.047 | .001* | .010** |
| Age 5 paternal psychological distress on age 3 externalising | -0.026 | 0.038 | .503 | .999 |
| Age 5 paternal psychological distress on age 3 maternal psychological distress | -0.143 | 0.056 | .011* | .103 |
| Age 5 paternal psychological distress on age 3 paternal psychological distress | 0.079 | 0.068 | .250 | .999 |
| *Note.* *significant at *p*<.05 (unadjusted). **significant at *q*<.05 (adjusted). Ages correspond to children’s median ages at data collection. | | | | |

**References**

Flouri, E., Sarmadi, Z., & Francesconi, M. (2019). Paternal Psychological Distress and Child Problem Behavior From Early Childhood to Middle Adolescence. *Journal of the American Academy of Child and Adolescent Psychiatry*, *58*(4), 453–458. https://doi.org/10.1016/j.jaac.2018.06.041
